# The Influence of Accelerated UV-A and Q-SUN Irradiation on the Antibacterial Properties of Hydrophobic Coatings Containing *Eucomis comosa* Extract

**DOI:** 10.3390/polym10040421

**Published:** 2018-04-09

**Authors:** Małgorzata Mizielińska, Urszula Kowalska, Piotr Salachna, Łukasz Łopusiewicz, Michał Jarosz

**Affiliations:** 1 Center of Bioimmobilisation and Innovative Packaging Materials, Faculty of Food Sciences and Fisheries, West Pomeranian University of Technology Szczecin, Janickiego 35, 71-270 Szczecin, Poland; urszula.kowalska@zut.edu.pl (U.K); lukasz.lopusiewicz@zut.edu.pl (Ł.Ł.); michal.jarosz@zut.edu.pl (M.J.); 2 Department of Horticulture, West Pomeranian University of Technology, 3 Papieża Pawła VI Str., 71-434 Szczecin, Poland; piotr.salachna@zut.edu.pl

**Keywords:** coatings, *Eucomis comosa* extract, antibacterial, antimicrobial properties

## Abstract

The purpose of this research was to examine the antimicrobial properties against Gram-positive bacteria, as well as the water vapour characteristic of polylactic acid (PLA) films covered with a methyl–hydroxypropyl–cellulose (MHPC)/cocoa butter carrier containing *Eucomis comosa* extract as an active substance. The second purpose of the study was to evaluate the influence of accelerated UV-A and Q-SUN irradiation (UV-aging) on the antimicrobial properties and the barrier characteristic of the coatings. The results of the study revealed that MHPC/cocoa butter coatings had no influence on the growth of *Staphylococcus aureus*, *Bacillus cereus*, and *Bacillus atrophaeus*. MHPC/cocoa butter coatings containing *E. comosa* extract reduced the number of bacterial strains. MHPC/cocoa butter coatings also decreased the water vapour permeability of PLA. It was shown that accelerated UV-A and Q-SUN irradiations altered the chemical composition of the coatings containing cocoa butter. Despite the alteration of the chemical composition of the layers, the accelerated Q-SUN and UV-A irradiation had no influence on the antimicrobial properties of *E. comosa* extract coatings against *S. aureus* and *B. cereus*. It was found that only Q-SUN irradiation decreased the coating activity with an extract against *B. atrophaeus*, though this was to a small degree.

## 1. Introduction

The food packaging industry is seeking to replace synthetic polymers with natural and biodegradable materials that present grease, water vapour, and gas barrier properties. Polylactic acid (PLA) is a compostable and renewable biopolymer that has been commercialized as a good alternative substitute for synthetic polymers [1,2,3,4,5,6]. It can be produced from the bacterial fermentation of renewable resources, such as sugar beet or corn starch. PLA has been approved as safe by the United States Food and Drug Administration (FDA). Polylactic acid has an advantage, as its physical and mechanical properties can be easily changed and tailored by simply varying its chemical composition (quantities of d- and l-isomer), as well as altering any processing conditions. It is also important that this biopolymer is commercially available, and its price is low [6]. As a consequence, these advantages give PLA the greatest potential for packaging and medical applications. 

With respect to barrier behaviour, PLA shows high gas transmission and low water vapour barrier that renders this polymer unsuitable for several food packaging applications in the case of synthetic polymers. To improve barrier characteristics, the surface of the polymer should be modified. A covering of biopolymer with coatings might be a solution [2,3,4,5,6,7]. For example, paraffin wax emulsions and polyurethane- or styrene-based copolymers are typical hydrophobic sizing agents that are applied in molten form to the surface of biopolymers and offer an improvement in water vapour barrier property. The purpose of the wax is to provide a moisture barrier. However, paraffin wax has relatively poor durability and flexibility as a surface-coating material. The addition of synthetic polymers and modifiers, such as cellulose derivatives, rubber derivatives, vinyl copolymers, polyamides, polyesters, and butadiene–styrene copolymers, compatible with wax, may overcome its defects. Often, special resins or plastic polymers can be added to the wax to improve adhesion and low-temperature performance and to prevent cracking [8]. Commercial products such as Eurocryl 2080 (Cebra Chemie, GmbH, Bramsche, Germany), Exceval HR 3010 (Kuraray, Europe GmBH, Hattersheim am Main, Germany), Ecroprint RA 112 (Michelman, Ecronova Polymer GmbH, Recklinghausen, Germany), Ultralub (Keim Additec Surface, GmbH, Kirchberg, Germany), Aquacer 2650 (Byk, Wesel, Germany) or cocoa butter (which is used in the food industry) are used as hydrophobic carriers to cover packaging materials. This butter is considered the most important cocoa by-product, due to its physical and chemical characteristics, which offer highly valued functional properties in the food industry. The amount of cocoa butter and the fatty acid profiles depend on the growing conditions of the cocoa beans. In cocoa butter, fatty acids are organised as triacylglycerol (TAG), the majority of these TAGs being 2-oleyl glycerides (O) of palmitic (P) and stearic (S) acids (POP, POS, SOS). Due to its hydrophobic properties, a cocoa butter carrier can be used to improve the water vapour barrier properties of PLA [9]. Unfortunately, cocoa butter is not compatible with PLA, which is a polyester. The addition of methyl–hydroxypropyl–cellulose, cellulose derivative, may improve in coating adhesion to the PLA film. 

In recent years, a great deal of effort has been devoted not only to barrier properties, but also to the development of coatings with antimicrobial activity that decreases bacterial growth. Attention has been concentrated on the development of coatings offering the highest possible eradication activity in the shortest possible time. For antibacterial coatings, long lifetime is a key requirement in many practical applications when these coatings are deposited on the contact surfaces of substrates, and must simultaneously perform two functions: antibacterial and protective [4,5,6,7,10,11,12,13]. The contact of active materials with foodstuffs, offering the ability to change composition or the atmosphere around it, is an active packaging system that inhibits or decreases the growth of microorganisms present on the surface of these perishable goods. Coatings with antimicrobial properties may contain an active substance e.g., zinc oxide (ZnO) nanoparticles, essential oils, organic acids, bacteriocins, exopolysaccharides, or plant extracts [4,5,6,7,10,11,12,13,14,15,16,17]. 

*Eucomis* (L.) L’Hér (Asparagaceae, formerly Hyacinthaceae) is a small genus consisting of bulbous geophytes extensively used in southern African traditional medicine [18,19,20,21,22]. Various plant parts and extract solvents of various *Eucomis* species have been tested for in vitro and in vivo antimicrobial screening [18,22,23,24,25]. It has been shown that *Eucomis* extracts inhibited *Bacillus subtilis*, *Escherichia coli*, and *Staphylococcus aureus* [22,23,24,25], as well as fungal cells, such as *Candida albicans* [24]. It was proven that *E. comosa* water extracts had a marked influence on the viability of the *S. aureus* strain in a previous study. A medium containing the *E. comosa* water extract caused a 2-log decrease in the number of bacterial cells on average. A decrease in the number of *S. aureus* was dependent on the extract concentration and was confirmed in tests, with the highest results being found in 25% extracts. It was also noted that *E. comosa* water extracts triggered a decrease in the viability of the *B. atrophaeus* strain [25,26]. 

The coating, which contained cocoa butter as a hydrophobic carrier, could be used to cover PLA films. The cocoa butter was able to increase the water vapour barrier of PLA. In addition, *E. comosa* extract as an active substance could be introduced into an MHPC/cocoa butter carrier to create antimicrobial coating activity. Boxes covered with Methocel™ containing polylysine and boxes containing polyethylene (PE) films covered with MHPC with ZnO nanoparticles were used as packaging material for fresh cod fillets [27]. It was demonstrated that the number of bacterial cells stored in boxes covered with Methocel™ containing polylysine or in the boxes containing the PE films coated with MHPC with ZnO nanoparticles did not go over 10^7^ cfu/g. Quite opposite results were obtained for boxes that were not covered with the active coatings (control samples). These results suggested that the active coatings improved the quality of cod fillets after storage. In marine fish stored in refrigerated aerobic conditions, *Pseudomonas* sp. and *Shewanella* spp. (Gram-negative bacteria) were observed as dominant. It was proved that MHPC coatings with ZnO nanoparticles [28] and polylysine [27] were active against Gram-negative bacteria, and they could be chosen as a packaging material to extend the quality and freshness of cod fillets after storage. The *Eucomis* extracts were active against *Bacillus* sp. and *Staphylococcus aureus* [25,26]. The polymer films or boxes covered with active coatings containing *E. comosa* extract could be used as packaging material for vacuum packed raw meat, fish, or cheese. The active coatings could extend the shelf life, quality, and freshness of food products. 

In general, an active packaging material should function during storage to inhibit microorganism growth and extend the shelf life of any given food product. This means that coatings should offer sufficient resistance against UV radiation [28]. Ultraviolet (UV) radiation is a section of the non-ionizing region of the electromagnetic spectrum that comprises of approximately 8–9% of total solar radiation. It can lead to a degradation in the physical–mechanical, optical, and antimicrobial properties of materials. Introducing an active substance that is sensitive to UV in a coating carrier can lead to coating inactivation after UV-aging. Introducing an active substance that is resistant to UV in a coating carrier can prevent the inactivation of this coating after UV-aging [28,29,30,31,32]. 

The aim of this research was to examine the antimicrobial properties against Gram-positive bacteria, as well as the water vapour characteristic of PLA films covered with an MHPC/cocoa butter carrier containing *E. comosa* extract as an active substance. The second aim of the study was to evaluate the influence of accelerated UV-A and Q-SUN irradiation (UV-aging) on the antimicrobial properties and the barrier characteristic of the coatings.

## 2. Materials and Methods

### 2.1. Materials

The test microorganisms used in this study were obtained from a collection from the Leibniz Institute DSMZ (Deutsche Sammlung von Mikroorganismen und Zellkulturen, Braunschweig, Germany). The strains were supplied from an American Type Culture Collection (ATCC, Manassas, VA, USA). The organisms used in this study were *S. aureus* DSMZ 346, *B. cereus* ATCC 14579, and *B. atrophaeus* DSM 675 IZT.

Polylactide films, (A4, 20 μm) (CBIMO—Center of Bioimmobilisation and Innovative Packaging Materials, Szczecin, Poland) were used in this research. MHPC (Chempur, Piekary Śląskie, Poland) was used as a coating carrier. *E. comosa* bulbs (Department of Horticulture, Szczecin, Poland) were used to prepare a water extract (as an active substance). To verify the antimicrobial properties of any coatings, agar-agar, TSB, and TSA mediums (Merck, Darmstadt, Germany) were used. All mediums were prepared according to the Merck protocol (each medium was weighed according to the manufacturer’s instructions, then suspended in 1000 mL of distilled water and autoclaved at 121 °C for 15 min).

### 2.2. Extract Preparation

*E. comosa* dried bulbs were ground to powder and a sample of 5 g was extracted with 50 g of 70% aqueous acetone. The sample was then kept in a sonication water bath for one hour. The temperature of the bath was maintained at 15 °C by adding ice. The acetone extract was concentrated at 40 °C. After the evaporation of the acetone, the sample was filtered through a 0.2 µm filter. The resulting 15 g of extract was used in further analyses.

### 2.3. Coating Preparation and Antimicrobial Properties Analysis

(1) MHPC (2 g) was introduced into 48 g of water, this was mixed for 1 h using a magnetic stirrer (Ika, Staufen im Breisgau, Germany) at 40 °C at 1500 rpm. Cocoa butter (10 g) was heated to 40 °C. Then, 40 g of MHPC was mixed with 10 g of cocoa butter and homogenized (1000 rpm) (Heidolph, Sigma-Aldrich, Poznań, Poland). The hydrophobic mixture was used to cover the PLA films to obtain coatings devoid of any active substances.
(2) *E. comosa* extract (12.5 g) was mixed with 25.5 g of water. Next, 2 g of MHPC was introduced into 38 g of *E. comosa* solution. The mixture was mixed for 1 h using a magnetic stirrer (Ika, Staufen im Breisgau, Germany) at 40 °C at 1000 rpm. Cocoa butter (10 g) was heated to 40 °C and 40 g of MHPC containing *E. comosa* extract was then mixed with the cocoa butter and homogenized (1000 rpm) (Heidolph, Sigma-Aldrich, Poznań, Poland). The hydrophobic mixture was used to cover the PLA films to obtain 25% active substance coatings.
(3) MHPC (2 g) was introduced into 48 g of water. The mixture was mixed for 1 h using a magnetic stirrer (Ika, Staufen im Breisgau, Germany) at 40 °C at 1500 rpm. The hydrophilic MHPC was used to cover PLA films to obtain coatings devoid of any active substances and hydrophobic substances. The films covered with MHPC were used for the analyses of Fourier transform infrared (FT-IR) and water vapour transmission rate (WVTR).

PLA films were covered using Unicoater 409 (Erichsen, Hemer, Germany) at a temperature of 40 °C with a 40 μm diameter roller. The coatings were dried for 2 h at a temperature of 25 °C. Layers (1.6 g) of MHPC/cocoa butter (1.28 g of MHPC and 0.32 g of cocoa butter) per 1 m^2^ of PLA were obtained. PLA films that were not covered were used as control samples (K). PLA films with MHPC/cocoa butter coatings were also used as control samples (MHPC/CB). 

The film samples were cut into square shapes (3 cm × 3 cm). The antimicrobial properties of non-covered and covered films were carried out according to the ASTM E 2180-01 standard [33]. As the first step of the experiments, *S. aureus*, *B. cereus*, and *B. atrophaeus* cultures originated from 24 h growth (coming from stock cultures) were prepared. The concentrations of the cultures were standardized to 1.5 × 10^8^ cfu/mL. The concentration of each culture was measured using Cell Density Meter (WPA, Cambridge, UK. CB4 OF J). The agar slurry was prepared by dissolving 0.85 g of NaCl and 0.3 g of agar-agar in 100 mL of deionized water and autoclaved for 15 min at 121 °C and equilibrated at 45 °C (one agar slurry was prepared for each strain). One millilitre of the culture (separately) was placed into the 100 mL of agar slurry. The final concentration of each culture was 1.5 × 10^6^ cfu/mL in molten agar slurry. The square samples of each film (not covered PLA films, covered PLA film, irradiated, and not irradiated) were introduced (separately) into the sterile Petri dishes with a diameter of 55 mm. Inoculated agar slurry (1.0 mL) was pipetted onto each square sample. The samples were incubated 24 h at 30 °C with relative humidity at 90%. After incubation, the samples were aseptically removed from the Petri dishes and introduced into the 100 mL of TSB. The samples were sonicated 1 min in the Bag Mixer^®^ CC (Interscience, St Nom la Brètech, France). The sonication facilitated the complete release the agar slurry from the samples. Then serial dilutions of the initial inoculum were performed. Each dilution was spread into the TSA and incubated at 30 °C for 48 h. The results were presented as an average value with standard deviations. 

### 2.4. Accelerated Irradiation

The non-covered and covered film samples were cut into rectangle shapes (23.5 cm × 7.0 cm and 26.0 cm × 2.5 cm) respectively. The samples were introduced into a UV-A accelerated weathering tester with 1.55 W/m^2^ (QUV/spray, Q-LAB, Homestead, FL, USA) and into Q-SUN accelerated Xenon Test Chamber with 1.5 W/m^2^ (Model Xe-2, Q-LAB, Homestead, FL, USA) and irradiated for 24 h [34].

### 2.5. FT-IR

Fourier transform infrared (FT-IR) spectrum of the non-covered and covered film samples was measured using a FT-IR spectroscopy (Perkin Elmer Spectrophotometer, Spectrum 100, Waltham, MA, USA), operated at a resolution of 4 cm^−1^ and four scans. Film samples were cut into square shapes (2 cm × 2 cm) and placed directly at the ray-exposing stage. The spectrum was recorded at a wavelength of 650–4000 cm^−1^.

### 2.6. Barrier Characteristic

Water vapour transmission rate (WVTR) was performed according to DIN 53122-1 [35] and ISO 2528:1995 [36]. WVTR was measured by means of a gravimetric method that is based on the sorption of humidity by calcium chloride and a comparison of sample weight gain. Initially, the amount of dry CaCl_2_ inside the container was 9 g. The area of PLA film (covered or not covered) was 8.86 cm^2^. Measurement was carried out for a period of 4 days, and each day, the containers were weighed to determine the amount of absorbed water vapour through the films. The results were expressed as average values from each day of measurement and each container. Analyses were carried out at 6 independent containers (6 repetitions) for each type of PLA films (covered with the coatings and not covered), calculated as a standard unit g/m^2^·h, and presented as a mean ± standard deviation. 

### 2.7. Statistical Analysis 

The statistical significance was determined using an analysis of variance (ANOVA) followed by Duncan’s test. This test was used to determine significant differences between numbers of the bacterial cells. The values were considered as significantly different when *p* < 0.05. All analysis was performed with Statistica version 10 (StatSoft, Kraków, Poland).

## 3. Results

### 3.1. Antimicrobial Properties

**Figure 1:**
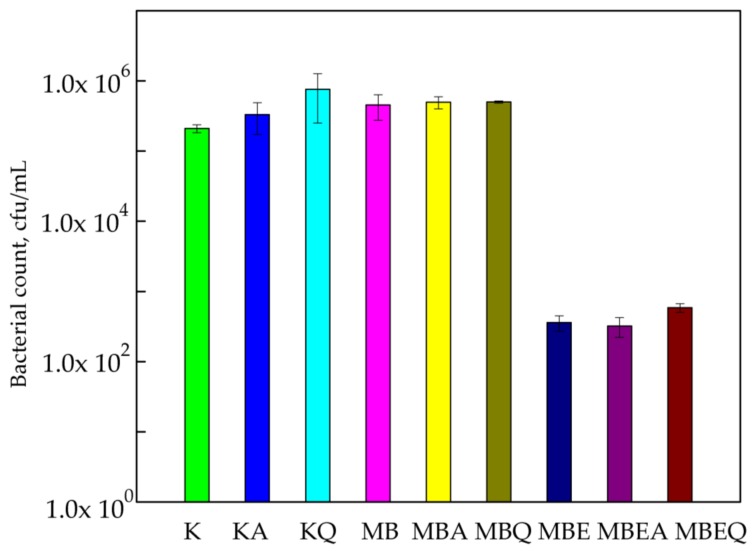
The influence of coatings on *S. aureus* growth. K—polylactic acid (PLA) film; KA—UV-A irradiated PLA film; KQ—Q-SUN irradiated PLA film; MB—PLA film, covered with methyl–hydroxypropyl–cellulose (MHPC)/cocoa butter coating; MBA—UV-A irradiated PLA film, covered with MHPC/cocoa butter coating; MBQ—Q-SUN irradiated PE film, covered with MHPC/cocoa butter coating; MBE—PLA film, covered with MHPC/cocoa butter coating, containing 25% of *E. comosa* extract; MBEA—UV-A irradiated PLA film, covered with MHPC/cocoa butter coating, containing 25% of *E. comosa* extract; MBEQ—Q-SUN irradiated PLA film, covered with MHPC/cocoa butter coating, containing 25% of *E. comosa* extract.

Study results indicated that MHPC coatings containing cocoa butter as hydrophobic additive had no influence on the growth of *S. aureus* cells. It was demonstrated that the number of *S. aureus* cells for the PLA film (control sample) was 2.1 × 10^5^ cfu/mL. The amount of the bacterial cells for the MHPC coating containing cocoa butter was similar compared to the control sample (4.56 × 10^5^ cfu/mL). The accelerated Q-SUN and UV-A irradiation did not influence the antimicrobial properties of the coatings devoid of bulb extract, while the MHPC coatings containing *E. comosa* extract inhibited the growth of *S. aureus.* The 3-log reduction of the number of *S. aureus* was noticed for the samples containing bulb extract (the number of the cells was 3.60 × 10^2^ cfu/mL). The *E. comosa* bulb extract demonstrated antimicrobial activity against Gram-positive bacteria, a point confirmed in a previous study [25,26]. Statistical analysis showed that the decrease in the number of bacterial cells was significant (*p* < 0.05). The accelerated UV-A irradiation did not change the antimicrobial properties of the coatings with bulb extract (3.2 × 10^2^ cfu/mL). In the case of Q-SUN irradiation (Figure 1), the number of bacterial cells marginally increased (5.86 × 10^2^ cfu/mL), later confirmed by a Duncan test (*p* > 0.05). 

**Figure 2:**
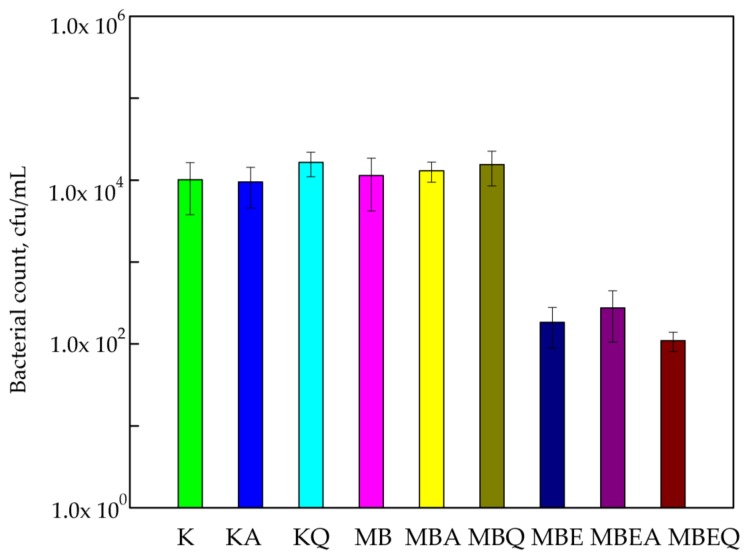
The influence of coatings on *B. cereus* growth. K—PLA film; KA—UV-A irradiated PLA film; KQ—Q-SUN irradiated PLA film; MB—PLA film, covered with MHPC/cocoa butter coating; MBA—UV-A irradiated PLA film, covered with MHPC/cocoa butter coating; MBQ—Q-SUN irradiated PE film, covered with MHPC/cocoa butter coating; MBE—PLA film, covered with MHPC/cocoa butter coating, containing 25% of *E. comosa* extract; MBEA—UV-A irradiated PLA film, covered with MHPC/cocoa butter coating, containing 25% of *E. comosa* extract; MBEQ—Q-SUN irradiated PLA film, covered with MHPC/cocoa butter coating, containing 25% of *E. comosa* extract.

The susceptibility assay of *B. cereus* with respect to the active coatings containing *E. comosa* extract is shown in Figure 2. Comparing the numbers of *B. cereus* cells for PLA films (1.01 × 10^4^ cfu/mL) to the numbers the *B. cereus* for PLA films covered with MHPC (1.14 × 10^4^ cfu/mL), it should be said that the numbers were almost the same. The results of this research determined that MHPC coatings containing cocoa butter were not found to be active against bacteria. Accelerated Q-SUN and UV-A irradiation did not influence the antimicrobial properties of the coatings devoid of any bulb extract, later confirmed by a Duncan test (*p* > 0.05). The marginally low change in the numbers of bacteria (lower than 1-log reduction) was noticed. The *B. cereus* cells exhibited sensitivity towards coatings containing *E. comosa* extract. The number of *S. aureus* decreased from 1.14 × 10^4^ to 1.84 × 10^2^ cfu/mL (2-log reduction). Statistical analysis showed that the decrease in the number of bacterial cells was significant (*p* < 0.05). Q-SUN and UV-A irradiation only marginally influenced the antimicrobial properties of coatings. UV-A irradiation deactivated the antimicrobial properties of the coatings with *E. comosa* extract. An increase in the number of bacterial cells (2.76 × 10^2^ cfu/mL) for these coatings irradiated with UV-A was also observed. In contrast to UV-A aging, Q-SUN improved the antimicrobial activity of the coatings (1.10 × 10^2^ cfu/mL). The differences between the numbers of viable cells were not significant, as later confirmed by Duncan’s test (*p >* 0.05).

**Figure 3:**
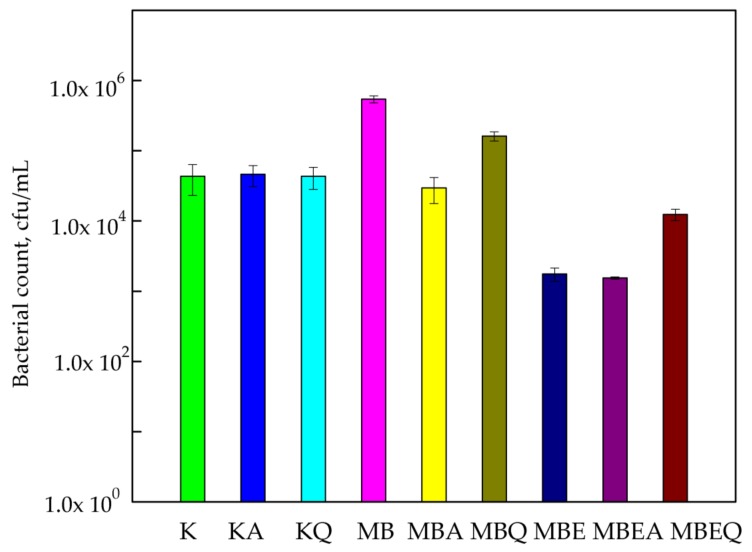
The influence of coatings on *B. atrophaeus* growth. K—PLA film; KA—UV-A irradiated PLA film; KQ—Q-SUN irradiated PLA film; MB—PLA film, covered with MHPC/cocoa butter coating; MBA—UV-A irradiated PLA film, covered with MHPC/cocoa butter coating; MBQ—Q-SUN irradiated PE film, covered with MHPC/cocoa butter coating; MBE—PLA film, covered with MHPC/cocoa butter coating, containing 25% of *E. comosa* extract; MBEA—UV-A irradiated PLA film, covered with MHPC/cocoa butter coating, containing 25% of *E. comosa* extract; MBEQ—Q-SUN irradiated PLA film, covered with MHPC/cocoa butter coating, containing 25% of *E. comosa* extract.

The results of this research demonstrated that MHPC coatings with cocoa butter had no influence on the decrease in *B. atrophaeus* cell growth. It was also observed that the number of bacterial cells (5.44 × 10^5^ cfu/mL) increased when compared to PLA films (4.34 × 10^4^ cfu/mL) that were not covered with coatings. It is tempting to suggest that a coating devoid of an active substance may be used by *B. atrophaeus* as a carbon source. It should be added that the increase in viable cells was significant, later confirmed by statistical analysis (*p* < 0.05). The growth of bacterial cells decreased after 24 h contact with MHPC coatings containing *E. comosa* extract from 5.44 × 10^5^ to 1.73 × 10^3^ cfu/mL. As seen below (Figure 3), the influence of accelerated UV-A irradiation on the antimicrobial properties of coatings with *E. comosa* extract was also not noted. In the case of Q-SUN irradiation, it was observed that the number of viable cells increased from 1.73 × 10^3^ to 1.24 × 10^4^ cfu/mL when compared to the non-irradiated samples. A statistical analysis demonstrated that the differences between numbers of *B. atrophaeus* cells were not significant (*p* > 0.05). 

### 3.2. FT-IR Analysis

PLA belonging to the polyester family has characteristic peaks. The infrared (IR) spectra at 2996.3 and 2946.49 cm^−1^ were assigned to the asymmetric and symmetric –CH stretching region of the –CH_3_ mode, respectively. The C=O stretching of the ester group was attributed as a broad and strong absorption bend at 1747.91 cm^−1^. The –CH_3_ bend was characterized by a peak of 1451.85 cm^−1^. –CH deformation and asymmetric bends were observed at 1381.82 and 1360.06 cm^−1^, respectively. The C=O stretching mode of the ester group were noted at 1266.41 cm^−1^ and an asymmetric C–O–C stretching mode were observed at 1180.81, 1127.28, and 1083 cm^−1^. In the region of 1000 and 800 cm^−1^, the bend at 955.57 cm^−1^ was attributed to the characteristic vibration of a helical backbone with CH_3_ rocking mode. Two bends related to the crystalline and amorphous phases of PLA were found at 868.06 (assigned to the amorphous phase), 766.57 and 755.32 cm^−1^ (crystalline phase). The results were confirmed by Chu Z. et al. [37] and Seda Tıglı Aydın R. et al. [37]. 

**Figure 4:**
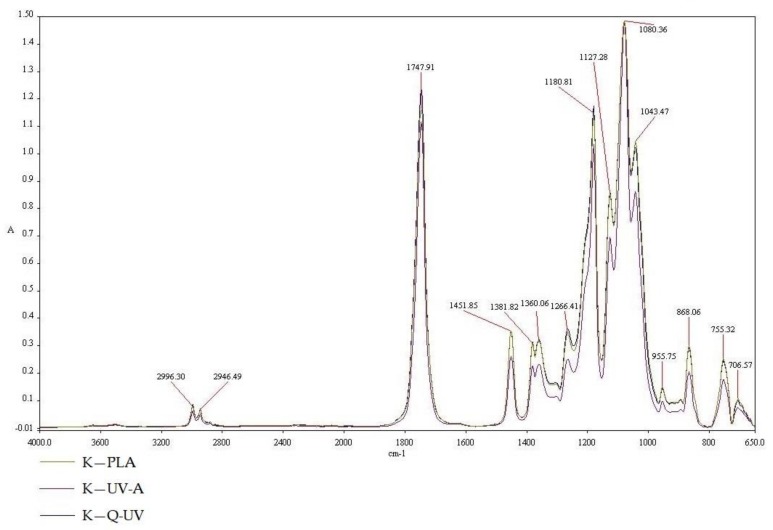
The FT-IR spectra of K—PLA, K—UV-A, K—Q-UV.

The influence of UV irradiation and Q-SUN irradiation on coatings can be clearly noted through the use of FT-IR spectroscopy. Properties that were found to influence the absorption peak and bend positions were; structure, chemical composition, as well as the morphology of thin films or coating [28,29]. Study results demonstrated that differences in chemical composition and morphology of the PLA films (K—PLA) after UV-A irradiation (K—UV-A) and Q-SUN irradiation (K—Q-UV) were not noted (Figure 4). As shown in Figure 4, the curves of irradiated PLA films were similar to the PLA film that was not irradiated. However, with the UV-aging of the samples, the bend, at 1381.82, 1360.06, and 868.06 cm^−1^, decreased in intensity. The results were confirmed by Yingfeng Z. et al. [38]. The authors demonstrated that the position of characteristic absorption peak of PLA did not change after aging treatment for different length of time, but significant change of the intensity of absorption peak was observed. The accelerated weather testing was performed also by Van Cong D. et al. [39] to evaluate the effects of TiO_2_ crystal forms on the degradation behaviour of an EVA/PLA/TiO_2_ nanocomposites compared to poly (ethylene-*co*-vinyl acetate) (EVA)/polylactic acid (PLA) blend. The results of FT-IR analysis, and thermal–mechanical properties confirmed the degradation of samples under accelerated weather testing. The degradation level of samples depended on TiO_2_ crystal forms present in samples. The TiO_2_ nanoparticles promoted the photodegradation of EVA/PLA/TiO_2_ nanocomposites, in which mixed crystals of TiO_2_ nanoparticles showed the highest photocatalytic activity. 

**Figure 5:**
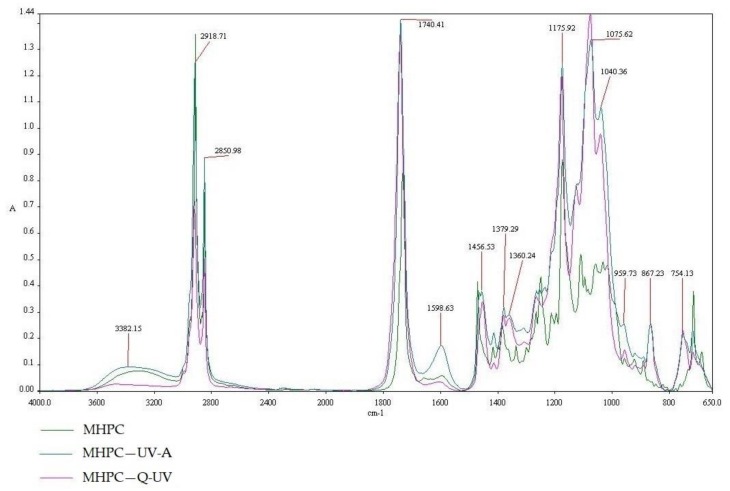
The FT-IR spectra of MHPC, MHPC—UV-A, MHPC—Q-UV.

A representative spectrum of MHPC is shown in Figure 5. The peak at 3382.15 cm^−1^ was due to –OH vibration stretching. The symmetric stretching mode of methyl and hydroxyl propyl frequency was found in the range of 2918.71–2850.98 cm^−1^ in which all the –CH bonds extend and contract in phase. Symmetric vibrations were mainly displayed in the range of 1379.29 cm^−1^ and suggested cyclic anhydrides. The bands at 1040.36 and 1075.62 cm^−1^ were for the stretching vibration of ethereal C–O–C groups. These results were confirmed by Punitha S. et al. [40] and by Dong Ch. et al. [41].

The results demonstrated that accelerated irradiation had no effect on PLA film samples. The influence of UV-A and Q-SUN irradiation on hydrophilic MHPC was also not observed (Figure 5). However, with the UV-aging of the samples, the bend, at 1381.82, 1360.06, and 868.06 cm^−1^, increased in intensity. 

**Figure 6:**
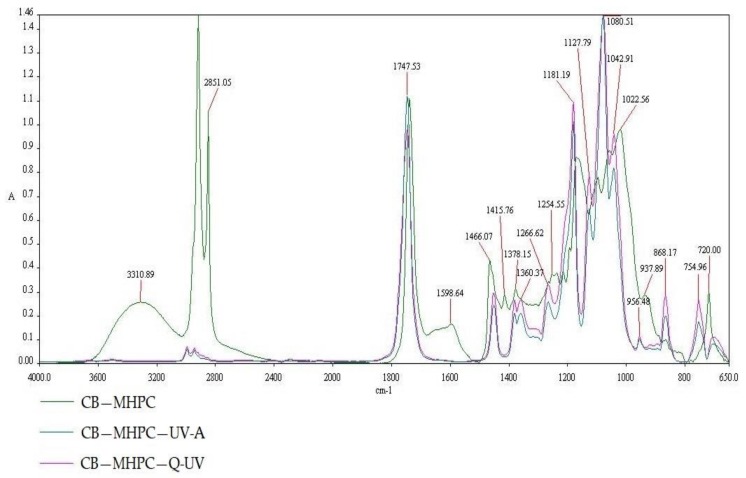
The FT-IR spectra of CB—MHPC, CB—MHPC—UV-A, CB—MHPC—Q-UV.

**Figure 7:**
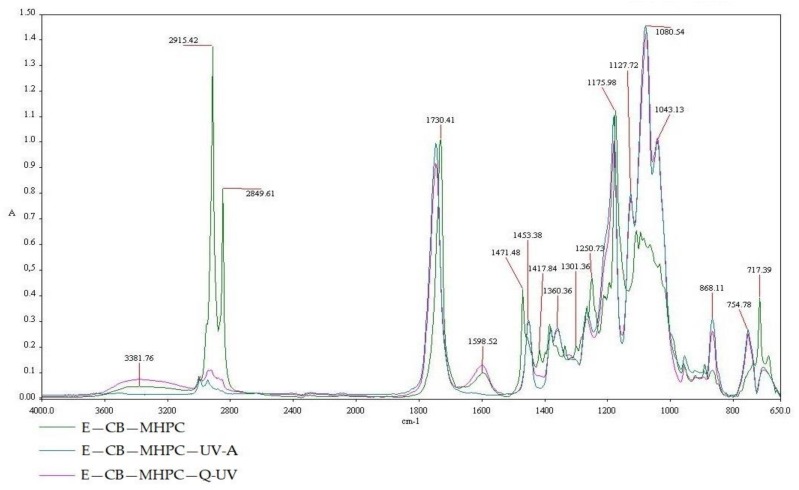
The FT-IR spectra of E—CB—MHPC, E—CB—MHPC—UV-A, E—CB—MHPC—Q-UV.

A MHPC coating containing cocoa butter (CB—MHPC), a UV-A irradiated MHPC coating with cocoa butter (CB—MHPC—UVA) and a Q-SUN irradiated MHPC coating with hydrophobic additive (CB—MHPC—Q-UV) are presented in Figure 6. There are three regions viewed in the FT-IR spectroscopy that range from (1) 3600 to 2400 cm^−1^; (2) 1800 to 1400 cm^−1^; and (3) 1000 to 650 cm^−1^. In the case of a 2851.05 cm^−1^ peak, this was noted and is consistent with absorption, stimulated by O=C–H double bonds. The 1747.53 cm^−1^ peak was stimulated by stretching vibration of carbonyl groups (C=O) from esters of triglycerides. The 1254.55 cm^−1^ peak was stimulated by C–O stretching vibration in ester. Alternatively, spectra peaks ranging from 1600 to 1400 cm^−1^, were observed for a peak with C=C induced absorption. These peaks were only observed for a coating that was not irradiated. UV-A and Q-SUN irradiations caused the peaks to disappear. Different peak properties were shown at 937.89 and 720 cm^−1^. The peaks were also observed in the case of a MHPC coating containing cocoa butter. The results were confirmed by Vesela A. et al. [42] and by Suparman et al. [43]. The presence of these peaks for a Q-SUN and an UV-A irradiated MHPC coatings was not noted. It was clearly confirmed that accelerated UV-A and Q-SUN irradiations altered the chemical composition of the CB—MHPC layer. It is tempting to suggest that accelerated irradiations cased the oxidation of the hydrophobic additive. Similar results were obtained for the MHPC coatings containing cocoa butter as a hydrophobic additive and *E. comosa* extract as an active substance (Figure 7). There are also three regions viewed in the FT-IR spectroscopy that range from (1) 3600 to 2400 cm^−1^; (2) 1600 to 1400 cm^−1^; and (3) 1000 to 650 cm^−1^. In the case of coatings containing an active extract, the disappearance of the peaks after accelerated irradiation was also noted. This means that accelerated irradiations caused an unsaturated bond oxidation of the cocoa butter fatty acids. This led us to believe that ZnO nanoparticles have shielding properties, in contrast to the *E. comosa* extract, which did not shield the coatings against accelerated irradiation. Mizielińsska et al. [28] showed that nano ZnO shielded the MHPC layer against Q-SUN irradiation. This conclusion was confirmed by El-Feky O.M. et al. [31] who used ZnO nanoparticles as an additive for coatings as a protection against UV irradiation.

### 3.3. Barrier Characteristic

The results of this study demonstrated that the MHPC coating had no significant influence on the barrier properties of PLA films. As clearly seen below (Table 1). The accelerated UV-A and Q-SUN did not improve and/or lower the water permeability of PLA covered with MHPC. Cocoa butter as a hydrophobic additive decreased the water permeability of coatings from 76.88 ± 1.24 (g/(m^2^ × h)) to 41.38 ± 3.45 (g/(m^2^ × h)). It was observed that contrary to UV-A irradiation, Q-UV irradiation improved the barrier properties of the coated PLA. The addition of *E. comosa* extract into the coating decreased the water permeability of the MHPC coating with cocoa butter. It should be mentioned that UV-A and Q-UV irradiations increased the water permeability of the PLA covered with MHPC containing cocoa butter as a hydrophobic substance and *E. comosa* extract as an active substance. 

## 4. Discussion

Pollution is increasing day by day, and environmental laws are becoming more stringent. Therefore, companies need to transform to meet their traditional objectives of cost reduction and good product quality, but also make efforts to implement a green and innovative set of technologies [44,45]. There is a need to replace synthetic polymers with natural and biodegradable materials such as polylactic acid. PLA is the most commercial biodegradable polymer, showing many advantages. Hence, different methods have been suggested in order to improve its chemical–physical performance and to obtain antimicrobial properties of the films. With respect to synthetic polymers, the barrier behaviour showed a high gas and water vapour transmission that rendered this polymer unsuitable for several food packaging applications. To improve barrier characteristics and activate some kind of antimicrobial effect, a surface polymer modification, such as a coating, would need to be created [2,7,8,9,10,11,12,13,16,17]. Coatings containing active substances can be divided into those that migrate into the packed product and those that do not. The active compounds should inhibit the growth of microorganisms responsible for packed product spoilage and pathogenic microorganisms. An active packaging containing plant and spice extracts or essential oils has antimicrobial properties against many bacteria and fungi. Their natural components include antimicrobial phenolic compounds, aldehydes, ketones, alcohols, ethers, and hydrocarbons. [10,11,16,17]. Because *E. comosa extract* [25,26] was found to be active against *S. aureus*, *B. cereus*, and *B. atrophaeus*, it was used as an active substance in the coatings. Due to the high water vapour permeability [13,16,17] of the PLA films or MHPC carrier, a hydrophobic additive was also introduced into the coatings. 

The results of the study showed that MHPC/cocoa butter coatings containing *E. comosa* extract reduced the number of *S. aureus*, *B. cereus*, and *B. atrophaeus* cells. The addition of cocoa butter into the coatings decreased the water vapour permeability of PLA. It was shown that accelerated UV-A and Q-SUN irradiations altered the chemical composition of the coatings containing cocoa butter. It was also observed that contrary to UV-A irradiation, Q-UV irradiation that caused the oxidation of the double bonds improved the barrier properties of the coated PLA. The addition of *E. comosa* extract into the coating decreased the water vapour permeability of the MHPC coating with cocoa butter. It should be mentioned that UV-A and Q-UV irradiations increased the water vapour permeability of covered PLA containing *E. comosa* extract. 

Despite alterations made to the chemical composition of the layers, accelerated Q-SUN and UV-A irradiation had no influence on the antimicrobial properties of *E. comosa* extract coatings against *S. aureus*, and *B. cereus* (in contrast to barrier characteristic). Only Q-SUN irradiation decreased coating activity with an extract against *B. atrophaeus*, but this was negligible. Similar results were obtained in a previous study [28] that showed that accelerated Q-SUN and UV-A irradiation had no influence on the antimicrobial properties of coatings against *S. aureus* and *B. cereus.* The coatings used in this previous study contained ZnO nanoparticles that shielded the active MHPC coating during irradiation, which was confirmed by the authors [31,46]. This work showed that *E. comosa* extract did not shield the MHPC/cocoa butter coating during irradiation. Although the extract had no shielding properties and the chemical composition changed, the coatings were still active against Gram-positive bacteria.

The growing demand for increased fresh food shelf life as well as the need of protection against foodborne diseases urged the development of antimicrobial food packaging. Active food packaging may include oxygen scavengers, moisture absorbers, ultraviolet barriers, or compounds that deliver flavouring, antioxidant, or antimicrobial agents. In the context of increasing demand of multiple hurdle technology to achieve high food safety standards, the development of antimicrobial packaging systems is of great interest. The use of natural products, such as essential oils (EOs) or plant extracts, as food preserving agents, is being promoted given the current trend towards green consumerism. These extracts or oils often contain compounds such as polyphenols or terpenes with antimicrobial properties [47,48,49,50,51,52,53,54]. Ginja cherry stem extracts, extracts from green tea were used in food packaging [50,51]. 

It is known that Gram-positive bacteria such as *S. aureus*, *Bacillus* sp., and *Listeria monocytogenes* were often isolated from raw fish, meat, or ready-sliced ham and cheese. Responsible for foodborne diseases, *Clostridium* sp. was isolated from meat packed in vacuum. The packaging materials covered with coatings, that are active against Gram-positive microorganisms could be the solution for vacuum system packaging. Shakila R.J. et al. [49] evaluated antimicrobial properties against *S. aureus* and *Listeria monocytogenes* of coatings with different active additives (chitosan, clove, and pepper) in vacuum packed fish steaks. The authors extended the shelf-life of product from 4 to 8 days at 4 °C. Sandoval L.N. et al. [54] proved that the active packaging containing chitosan as an antimicrobial agent offered an alternative for the preservation of the quality of fresh cheese during storage, and increased shelf life, and more importantly, it inhibited the growth of *L. monocytogenes*. This study showed that *E. comosa* extract coatings were active against Gram-positive bacteria even after UV-aging. These coatings could be used as packaging material for vacuum-packed fish, meat, or cheese.

## 5. Conclusions

The food packaging industry is going to replace non-compostable polymers with biodegradable materials. It should be underlined that polylactic acid is a biodegradable biopolymer that has been commercialized as a good alternative substitute for synthetic polymers. 

It should be underlined that the covering of PLA films with a MHPC carrier containing cocoa butter decreased water vapour permeability of the biopolymer. The additional advantage of the material was its antibacterial activity, obtained by introducing *E. comosa* extract as an active substance into the MHPC coating. Accelerated UV-A and Q-SUN irradiations altered the chemical composition of the coatings containing cocoa butter or cocoa butter and *E. comosa* extract. It caused an increase in the water vapour permeability of the coated PLA. It should be highlighted that the accelerated Q-SUN and UV-A irradiation had no influence on the antimicrobial properties of *E. comosa* extract coatings against *S. aureus* and *B. cereus*. 

Due to the resistance of the coatings to UV-aging, and due to improved barrier characteristic of PLA, the coatings could be used to cover biopolymer films or boxes to obtain the active packaging material. The active coatings could extend the shelf life, the quality and freshness of food products.

## Figures and Tables

**Table 1:** The water permeability of PLA films before and after irradiation.

Sample		WVTR (g/(m^2^ × h))
Devoid of irradiation	PLA	80.22 ± 1.78
	MHPC	76.88 ± 1.24
	CB—MHPC	41.38 ± 3.45
	E—CB—MHPC	29.91 ± 2.28
UV-A irradiated	MHPC	76.45 ± 0.25
	CB—MHPC	48.34 ± 4.68
	E—CB—MHPC	60.31 ± 1.02
Q-UV irradiated	MHPC	74.89 ± 2.76
	CB—MHPC	40.23 ± 3.49
	E—CB—MHPC	63.26 ± 1.44
